# Evolutionary Evidence for Alternative Structure in RNA Sequence Co-variation

**DOI:** 10.1371/journal.pcbi.1003152

**Published:** 2013-07-25

**Authors:** Justin Ritz, Joshua S. Martin, Alain Laederach

**Affiliations:** 1 Department of Biology, University of North Carolina, Chapel Hill, North Carolina, United States of America; 2 National Evolutionary Synthesis Center, Durham, North Carolina, United States of America; University of Missouri, United States of America

## Abstract

Sequence conservation and co-variation of base pairs are hallmarks of structured RNAs. For certain RNAs (e.g. riboswitches), a single sequence must adopt at least two alternative secondary structures to effectively regulate the message. If alternative secondary structures are important to the function of an RNA, we expect to observe evolutionary co-variation supporting multiple conformations. We set out to characterize the evolutionary co-variation supporting alternative conformations in riboswitches to determine the extent to which alternative secondary structures are conserved. We found strong co-variation support for the terminator, P1, *and* anti-terminator stems in the purine riboswitch by extending alignments to include terminator sequences. When we performed Boltzmann suboptimal sampling on purine riboswitch sequences with terminators we found that these sequences appear to have evolved to favor specific alternative conformations. We extended our analysis of co-variation to classic alignments of group I/II introns, tRNA, and other classes of riboswitches. In a majority of these RNAs, we found evolutionary evidence for alternative conformations that are compatible with the Boltzmann suboptimal ensemble. Our analyses suggest that alternative conformations are selected for and thus likely play functional roles in even the most structured of RNAs.

## Author Summary

RNA (Ribonucleic Acid) is a messenger of genetic information, master regulator, and catalyst in the cell. To carry out its function, RNA can fold into complex three-dimensional structures. Certain classes of RNAs, called riboswitches, adopt at least two alternative structures to act as a switch. We set out to detect the evolutionary signal for alternative structures in riboswitches as we hypothesize that these RNA sequences must have evolved to allow both conformations. We find that indeed such signals exist when we compare the sequences of riboswitches from multiple species. When we extend this analysis to other RNA regulators in the cell that are not thought of as switches, we detect equivalent evolutionary support for alternative structures. Viewed through the lens of evolutionary structure conservation RNA sequences appear to have adapted to adopt multiple conformations.

## Introduction

RNA is unique in that it is both a messenger of genetic information and it can fold to adopt highly specific functional conformations that carry out catalysis in the cell [1]–[4]. Large RNAs have a high propensity to misfold, requiring chaperones and in many cases protein co-factors to achieve an active conformation [5]–[7]. Riboswitches are a class of RNAs that must adopt at least two conformations to function, since it is ligand binding induced conformational change that allows them to regulate transcription and/or translation [8]–[12]. These molecules present an interesting evolutionary challenge since the sequence space should allow both conformations [13]–[16]. Furthermore, even small changes in sequence can significantly alter their structure and favor non-functional conformations [11], [17].

Co-variation of RNA bases across species is one of the strongest signals in biological sequences [18]–[21] and is observed when homologous sequences of RNAs are aligned [22]–[24]. The near perfect isostericity of the canonical G-C and A-U base-pairs results in their interchangeability in most RNA stems [25], [26]. For an RNA that adopts a single conformation to carry out its function (e.g. a group I intron), we expect that the ensemble of co-varying pairs should point to a single structure. For riboswitches, which must adopt at least two conformations we hypothesize that co-variation should be observed in alignments supporting both conformations.

**Figure 1:**
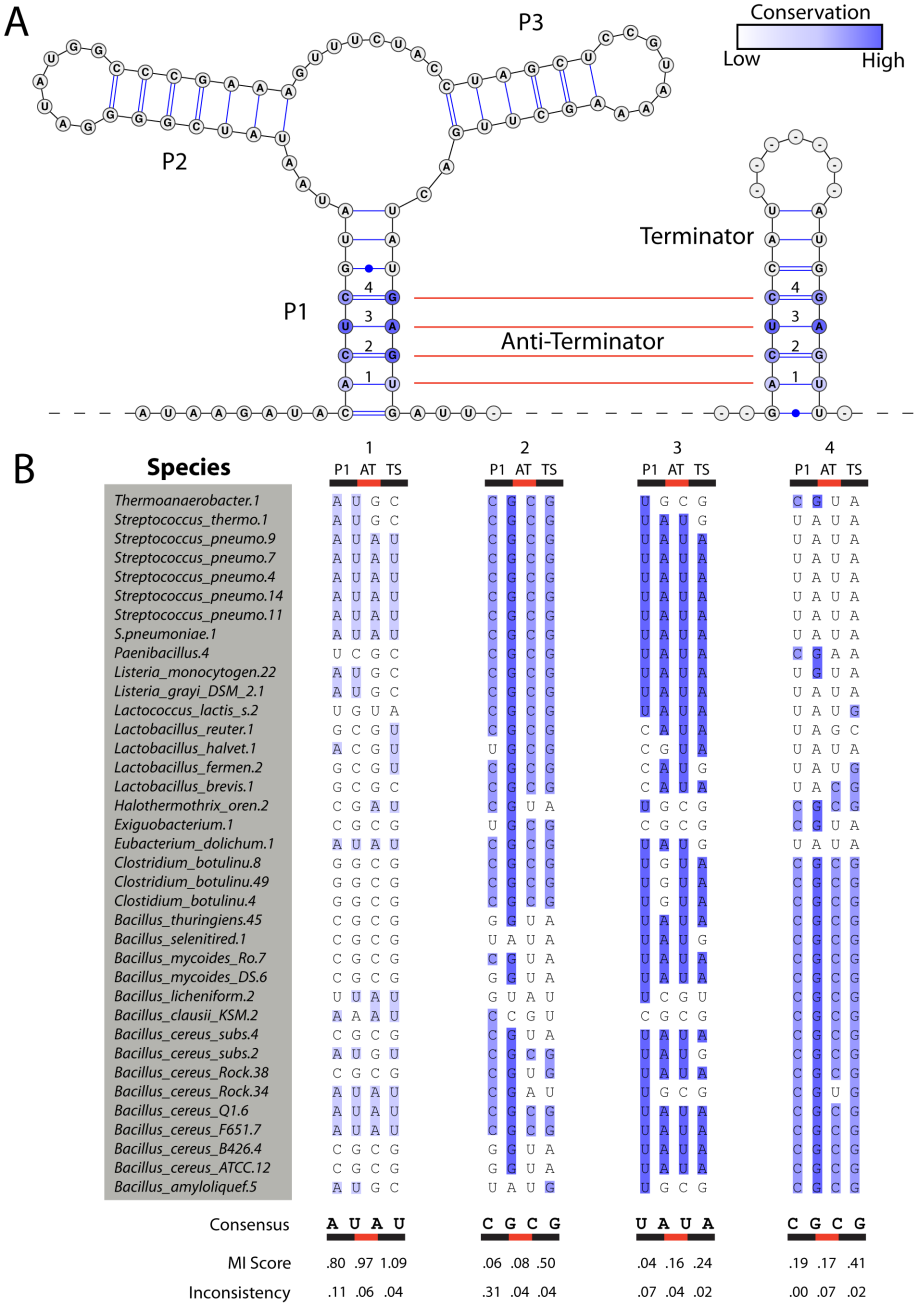
Co-variation analysis of purine riboswitch P1, terminator and antiterminator stems [9], [27], [28], [32]. A) Consensus secondary structure of the aptamer (P1, P2 and P3) and terminator domains. The anti-terminator base-pairs are indicated with red lines, and binding of the purine ligand to the aptamer domain alters the equilibrium between terminator and anti-terminator stems. B) Representative portion of RFAM RF00167 illustrating co-variation of base-pairs in the P1, terminator and antiterminator stems. We computed Mutual Information (MI) and inconsistency scores for each pair of columns in the alignment, considering only canonical Watson-Crick base pairs and G-U wobbles. We observe significant MI and inconsistency values for the antiterminator stem, indicating evolutionary evidence for alternative conformations in RNA.

The purine riboswitch is the simplest system in which we hypothesize it should be possible to observe co-variation supporting alternative conformations [27]–[29]. The system is schematically represented in Figure 1A and includes two domains (P1, P2, P3, which is the aptamer domain) and the terminator stem. We aim to determine the relative co-variation support for the P1, terminator *and* anti-terminator stems of the purine riboswitch to characterize the evolutionary signal for RNAs known to function through multiple conformations. Our analysis of this relatively simple conformational switch provides insight into the strength of the evolutionary signal that can be expected supporting multiple conformations. By then applying a similar analysis to other RNA alignments we aim to determine the likelihood of functionally important alternative conformations in structured RNA.

## Results

### Co-variation of the anti-terminator stem in the purine riboswitch

We begin our investigation into the evolutionary evidence supporting alternative conformations by considering an RNA that adopts at least two conformations to carry out its function. The purine riboswitch changes conformation in the presence of its' ligand (generally a purine base or derivative) to regulate protein biosynthesis [30]. Figure 1A represents the secondary structure of the “off” conformation for the consensus purine riboswitch as determined by crystallography [31]. The structure includes the characteristic P1, P2 and P3 stems of the purine riboswitch as well as the terminator hairpin, which has not been solved by crystallography [32], [33]. The mechanism of action of this riboswitch is particularly relevant to our study as it involves a significant secondary structure rearrangement. In the “on” state, the P1 stem is not base-paired; instead the anti-terminator is formed (indicated with red lines in Figure 1A). Given that both the “on” and “off” states of the riboswitch are functionally essential, one might expect to see co-variation in the anti-terminator base-pairs across species of purine riboswitches.

Co-variation models (CM) used to identify purine riboswitches in genomic sequences usually include only the aptamer domain. This is the case of Rfam family RF00167, which is the starting point of our analyses for this investigation [34]–[36]. To determine the level of co-variation support for the P1, terminator and anti-terminator stems in the purine riboswitch we aligned the 246 sequences from RFAM family RF00167 in which we could identify a terminator stem within 100 nucleotides of the aptamer domain [36], [37]. We then computed mutual information (MI) and inconsistency scores for the columns in the alignment corresponding to the three stems. These two scores evaluate the extent of co-variation between two columns in the alignment. A high MI score indicates that a specific base in one column is highly predictive of the base that is in the second column. A low inconsistency score indicates that in most cases the co-variation is between canonical base-pairs (G-C to A-U for example). The MIfold package was used to compute these scores considering only canonical and G-U base-pairs, which serves as the metric for co-variation of pairs of nucleotides in this study [18]. A base-pair with high MI and low inconsistency is therefore well-supported by evolutionary evidence. We find that the MI and inconsistency metrics are inversely correlated, as expected and this suggests that our results are mostly metric agnostic (see Figure S7). These data are often used to confirm and/or improve RNA secondary structure predictions [19], [21], [38]. In Figure 1B, the first AU base-pairs in both the P1 and Terminator Stem (TS) have high MI values, but we also observe equivalent evolutionary evidence (MI 0.97, inconsistency 0.04) for the anti-terminator (AT) pair. This is consistent with the RNA adopting multiple conformations when it acts as a ligand-induced switch. We observe similar trends for the three other P1/AT/TS pairs reported in Figure 1B. Additional MI and inconsistency values for the purine riboswitch are reported in Table S1 and confirm this trend. We therefore observe equivalent evolutionary support in the purine riboswitch alignment for the anti-terminator stem relative to the P1 and terminator stems.

### Alternative structures are revealed by Boltzmann suboptimal sampling

**Figure 2:**
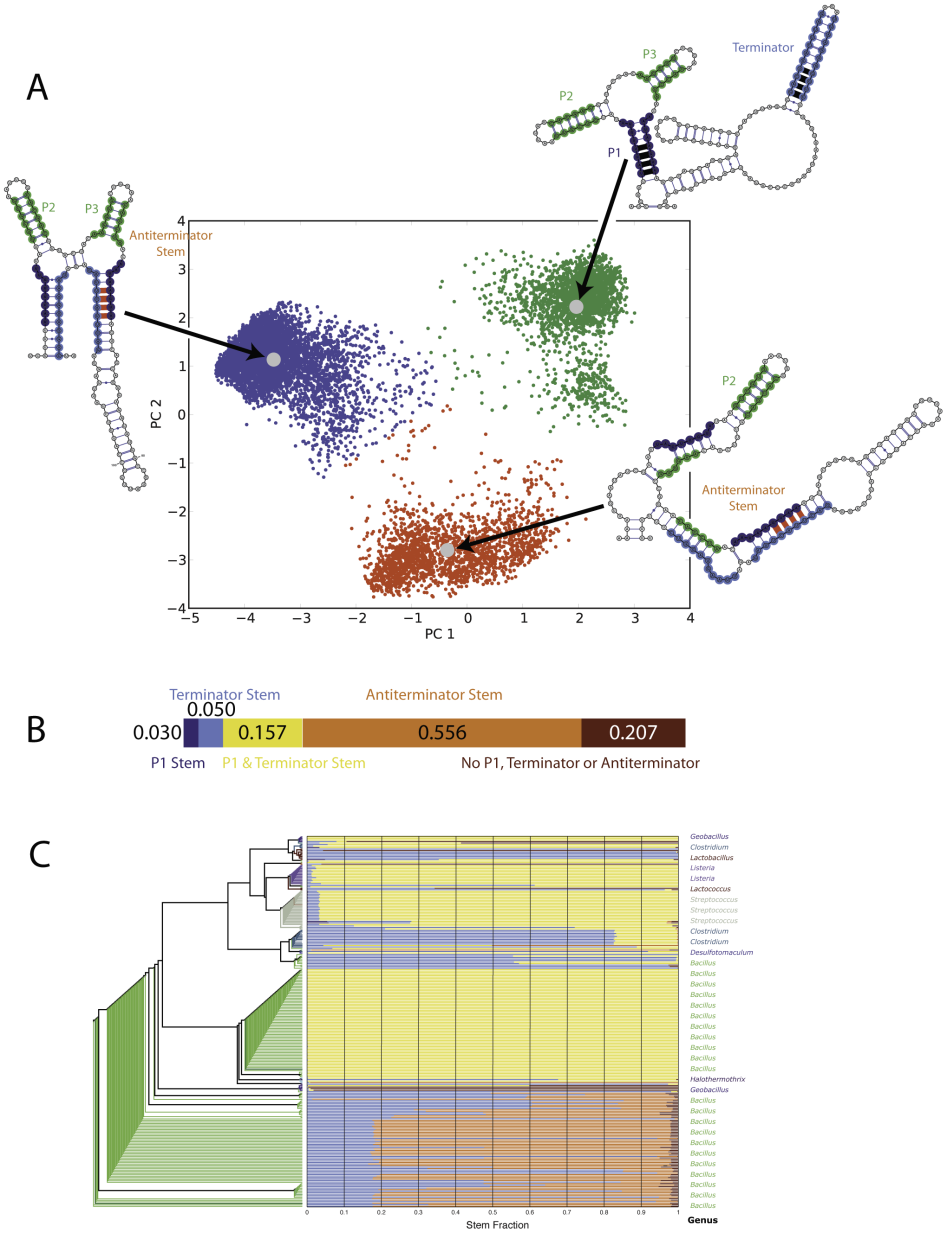
Structural analysis of the Boltzmann suboptimal ensemble of the purine riboswitch [40], [83]. A) Principal Component Analysis (PCA) of 10,000 suboptimal conformations of the *Streptococcus pneumoniae* purine riboswitch sequence reveals three major conformations, indicated in red, green and blue. The green structures predominantly include the canonical “off” structure of the riboswitch with formation of the terminator, P1, P2 and P3 stems. The red and green clusters predominantly include the “on” conformation with the antiterminator stem formed. B) Analysis of the relative abundance of the five possible conformations of the P1, terminator and antiterminator stem. This analysis reveals that the suboptimal ensemble is structurally diverse. C) Analysis of possible P1, terminator and antiterminator abundances for all purine riboswitch sequences organized according to their alignment tree. We observe that different classes of riboswitches partition differently, suggesting evolutionary adaptation of the multiple conformations of the riboswitch.

A riboswitch changes conformation upon ligand binding, allowing it to regulate transcription and/or translation [27]–[29]. To determine whether thermodynamic folding models support alternative conformations we performed Boltzmann suboptimal sampling of the *Streptococcus pneumoniae* purine riboswitch sequence, including the five single point mutations that most significantly affect structure as determined by SNPfold [11], [39]. Principal component analysis of 10,000 Boltzmann sampled suboptimal structures based on binary base-pair vectors reveals three major clusters of structures [11], [39], [40], with cluster probabilities of 20% (red), 56% (blue) and 24% (green). The “off” conformation of the riboswitch (Figure 2A, green cluster, the terminator stem is formed) as well as two alternative “on” conformations where the anti-terminator stem (red and blue clusters) are represented in the ensemble.

The high co-variation base-pairs identified in Figure 1 are highlighted in red (anti-terminator) and black (terminator, P1) in Figure 2A, indicating that the Boltzmann ensemble is consistent with the evolutionary analysis presented in Figure 1. Suboptimal sampling reveals that there are two classes of “on” conformations possible (red and blue clusters), which is not necessarily evident from the co-variation data alone. In addition, it is possible to classify each suboptimal structure into five mutually exclusive categories depending on the structural features present: P1 stem, terminator stem, P1 & terminator, Anti-terminator stem, and no P1, terminator or Anti-terminator stems. When we classify each of the 10,000 suboptimally sampled structures represented in Figure 2A, we see that a majority (55.6%) fall into the antiterminator stem category (Figure 2B). We also find that 15.7% of the structures have both P1 and the antiterminator stem formed (Figure 2B, yellow bar). Interestingly, 20.7% of the suboptimal structures adopt conformations where none of the characteristic riboswitch features are present (No P1, terminator or Anti-terminator).

These data suggest that although the principal component visualization used in Figure 2A suggests three major classes of structures, the RNA suboptimal ensemble is even more complex. This is borne out by the fact that only 37.6% of the variance of the structural ensemble is captured in the first two principal components. It is also due to the fact that the PCA space is determined by overall structural similarity and not the restricted analysis of the terminator, P1 and antiterminator stems reported in Figure 2B. In addition, we purposefully explored riboswitch conformational space by including select point mutants that increase structural diversity to generate the principal component space [11], [39].

We performed suboptimal sampling on all the purine riboswitch sequences previously identified from RFAM RF00167 that include a terminator sequence. We analyzed each ensemble identifying the P1 and terminator elements in the suboptimal structure and plotting their relative abundance using the same coloring scheme as in Figure 2B. We report these data projected onto the phylogenetic tree from our riboswitch alignment in Figure 2C. These data reveal a qualitative correlation between structural partitioning and phylogenetic origin of the riboswitch. Certain riboswitch sequences have evolved to adopt predominantly one structure (e.g. the top half of *Bacilli* riboswitches are predominantly “off” with P1 and terminator stems formed, while the *Desulfotomaculum* sequence is likely on, with predominantly the antiterminator stem formed). Thus, evolution appears to fine-tune the partitioning of the Boltzmann ensemble to favor specific conformations.

### High-MI alternative base-pairs in “structured” RNAs

The data presented in Figures 1 and 2 agree with our understanding of riboswitch function and the need for these RNAs to adopt at least two conformations to carry out their function. It is not surprising to find evolutionary evidence supporting alternative secondary structures in riboswitch alignments. However, catalytic ribozymes, such as the *Tetrahymena thermophila* group I intron must adopt a single structure to precisely organize the catalytic site and carry out their function [5], [41]–[47]. We might expect to see less evolutionary evidence for alternative RNA conformations in a “highly structured” family of RNAs like the group I introns [5], [48]–[50]. To test this hypothesis we performed an analogous MI analysis on the group I intron alignment from the Comparative RNA Web (CRW) database [51].

**Figure 3:**
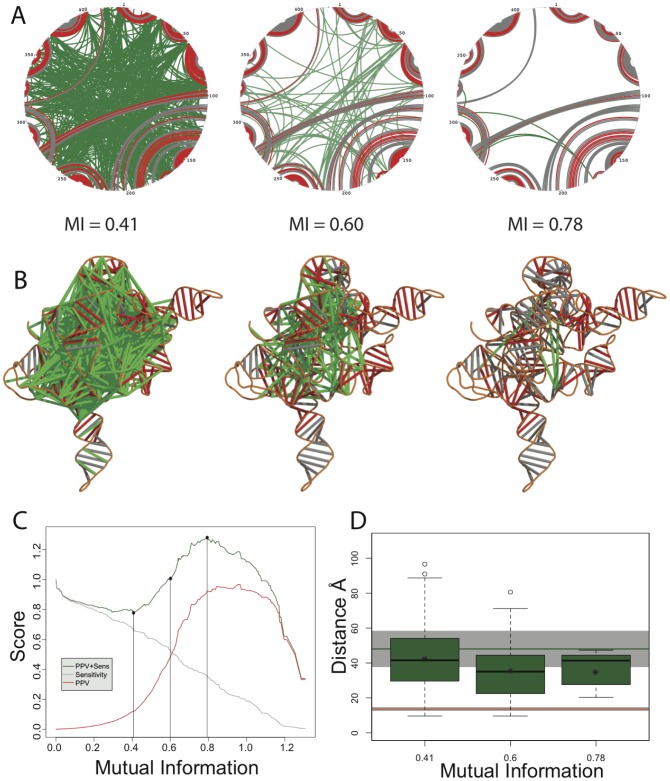
MI analysis of Watson-Crick base-pairing for the group I intron alignment from the Comparative RNA Website (CRW, [19, 51]). A) Circle diagrams of the L-21 *T. thermophila* group I intron structure, the secondary structure is derived from the crystal structure (PDB ID 1X8W) and is indicated in gray [52]. Base-pairs having MI support above the specified thresholds (MI = 0.41, 0.60 and 0.78) are colored red and those for which there is MI support above the threshold but that are not in the crystal structure are shown in green. B) Full length three-dimensional structural model of the *T. thermophila* group I intron derived from the crystal structure (helices missing in the crystal were modeled using the Nucleic Acid Simulation Tool (NAST) previously [53]. Base-pairs are projected onto the model using the same coloring scheme as in A). C) Sensitivity and PPV as a function of MI for the group I intron alignment. In this case sensitivity is computed as the number of “red” pairs divided by the total number in the accepted structure (gray pairs). PPV is computed as the number of True Positives (TP, in this case red pairs) divided by the sum of the TP and False Positives (FP, green pairs). We see that the sensitivity declines steadily with increasing, and that the data are maximally predictive at an MI value of 0.78, where the sum of PPV and sensitivity are maximal. Nonetheless, even in this case a significant number of co-varying canonical base-pairs (green) persist. D) Mean crystallographic distance in **Å** of non-accepted (green) base-pairs at the three MI thresholds. We see that at all three thresholds the means are above those of canonical base-pairs (shown as a red line at 18 **Å**), indicating that the co-variation is not likely the result of stabilizing an RNA tertiary contact.

Base-pairs with MI values above three different thresholds on a circle diagram representing the *T. thermophila* group I intron are illustrated in Figure 3A. The secondary structure derived from the crystal structure of the intron is represented in dark gray [52], base-pairs in the accepted structure with MI values greater than the threshold are indicated in red. Green base-pairs have MI values above the threshold but are not in the crystal structure [52]. In Figure 3B, the same coloring scheme is used to project high-MI canonical pairs onto a crystal structure informed model of the three-dimensional structure of the intron [53]. Visual inspection of the three-dimensional structure reveals that most of the high-MI pairs that are not secondary structure are long range, spanning a significant section of the 3D structural model [25], [54].

The green pairs illustrated in Figures 3A and 3B are “false-positives” in terms of the prediction of the crystal structure pairs. Extending this logic, red pairs are “true-positives,” and gray pairs are “false-negatives.” The rates of true, false, positives and negative vary with MI making it possible to compute sensitivity and Positive Predictive Value (PPV) as a function of the threshold. We computed PPV and sensitivity for these data and report the resulting curves in Figure 3C. The sum of PPV and sensitivity (green curve, Figure 3C) reveals the reason behind our choice of three MI values as illustrative thresholds, with an MI value of 0.41 representing the minima in PPV and Specificity, and the value of 0.78 the maximum.

The data plotted in Figure 3A clearly show strong MI evidence is found in the group I intron alignment for non-crystal base-pairs at all MI thresholds (green lines). It is important to point out that above the highest MI threshold (0.78) the sensitivity (gray line, Figure 3B) is 33%, i.e. a majority of crystal base-pairs are not supported by MI. The significant number of gray base-pairs in Figures 3A and 3B at the 0.78 threshold demonstrate that some of the stems are not supported by even a single co-varying base-pair. In their seminal determination of the *T. thermophila* intron structure, Michel and Westhof did not take into account the non-accepted (green) base-pairs for their structural model [38]. These are incompatible with a single structure model and have previously been considered false-positives for structure determination. In general, the paradigm for RNA structure prediction based on co-variation analysis has been to identify the structure that is compatible with the maximum number of co-varying pairs based on the idea that a specific RNA sequence folds to a single conformation [18], [19], [21].

Another explanation for the non-accepted base-pair co-variation observed in the group I intron alignment is non-canonical base-pairing and/or tertiary (3D) interactions [25], [54], [55]. We expect that if the green pairs in Figure 3A are due to short-range non-canonical 3D interactions, these nucleotides should be close in space in the crystal structure of the group I intron [52]. When these are projected onto the three-dimensional structure of the intron as in Figure 3B, these pairs are not close in three-dimensional space. The mean pair distances for all the pairs above the MI threshold that are not in the accepted structure is plotted in Figure 3D. As a reference the mean distance for accepted base-pairs in the structure (18 **Å**) is indicated as a red line (gray indicates ±1 standard deviation), while the mean pairwise distance for all pairs (48 **Å**) is indicated as a green line. We can see that at all MI thresholds, the green pairs are longer range than the expected 18 **Å** average of a canonical base-pair. It is therefore not likely that this evolutionary signal arises due to long-range tertiary contacts in the RNA.

### Ubiquitous long-range high-MI base-pairs in RNA alignments

We repeated the analysis performed on group I introns alignments for six other RNA families and summarize our findings in Table 1, Figures S1, S2, S3, S4, S5, S6 include analogous PPV an sensitivity plots for these RNAs. High-MI, low inconsistency pairs are found in all the RNAs we studied that are incompatible with the crystal structure in approximately the same proportion as what we observed with the purine riboswitch and group I intron alignments. More importantly, it is not possible to discern between RNAs that are generally thought to adopt a single conformation (e.g. tRNA) and multiple conformations (e.g. riboswitches). Effectively, when viewed through the lens of co-variation, all RNAs are the same in terms of their propensity to evolve alternative conformations.

**Table 1:** Summary statistics for structural analysis of high-MI base-pairs.

	Length/PDBID/RFAM/CRW	PPV	Sens.	PPV Shuff.	Sens. Shuff.	Mean Distance - Å
**Group I Intron**	421					13.77 (2.21)
0.41	1GRZ (1X8W)	0.128	0.636	0.199	1	42.03 (16.82)
0.595	IC1	0.464	0.522	0.872	1	36.11 (15.79)
0.78		0.906	0.364	0.962	0.392	36.24 (11.67)
**Purine RS**	111					13.63 (.60)
0.50	1Y26	0.154	0.300	0.483	1	27.21 (13.72)
0.705	RF00167	0.467	0.233	0.882	0.536	45.07 (-)
0.91		0.833	0.167	0.75	0.214	-
**Phe-tRNA**	76					13.50 (.55)
0.03	1EHZ	0.048	0.520	0.077	1	31.01 (13.02)
0.23	(F) [Type 1]	0.317	0.520	0.5	1	24.47 (9.67)
0.43		0.917	0.440	0.846	0.524	27.95 (-)
**16s**	1487					13.57 (.60)
0.40	1FJG	0.056	0.219	0.252	1	82.54 (40.85)
0.635	Bacterial rRNA	0.230	0.150	0.994	0.663	74.79 (37.62)
0.87		0.921	0.075	0.95	0.083	77.73 (103.90)
**Group II Intron**	173					13.50 (.48)
0.09	3G78	0.033	0.814	0.041	1	42.08 (18.35)
0.30	RF02001	0.395	0.698	0.56	1	33.01 (15.32)
0.51		0.913	0.488	0.917	0.524	22.28 (12.17)
**Glycine RS**	88					13.60 (.34)
0.11	3OX0	0.106	0.630	0.175	1	34.35 (13.90)
0.315	RF00504	0.667	0.481	0.903	0.686	37.39 (18.71)
0.52		0.900	0.333	0.818	0.333	44.90 (-)
**CID-GMP RS**	99					12.97 (1.61)
0.22	3IRW	0.110	0.346	0.329	1	26.19 (11.32)
0.395	RF01051	0.514	0.327	0.892	0.635	22.47 (12.42)
0.57		0.636	0.269	0.833	0.385	14.27 (-)

### Column shuffling yields similar fractions of putative alternative high-MI pairs

We performed column shuffling on the alignments using the RNAz “rnazRandomizeAln” algorithm to determine the expected number of alternative high-MI pairs [56], [57]. The RNAz algorithm is designed to maintain local conservation patterns by only shuffling columns in the alignment with similar degrees of conservation [24], [57]. One limitation of this approach is that no crystal structure exists as a standard for identifying long-range high-MI base-pairs. Furthermore, RNAAlifold predictions based on the shuffled alignment result in sparsely paired RNAs [18], [58]. We therefore generated a reference structure by considering all base-pairs above a threshold MI so as to have an equivalent number of pairs in the reference as in the crystal. Using this reference we computed expected PPV and sensitivity values for each shuffled alignment and report these in Table 1. Our data indicate similar PPV and sensitivity values to those computed using non-shuffled alignments in the previous section, albeit with on average slightly higher sensitivity for lower MI thresholds. These results suggest that the evolutionary process does not necessarily select for or against multiple conformations but instead tolerates these from those that are expected by chance.

## Discussion

From a chemical perspective, RNA is one of the simplest biopolymers in the cell being composed of purine and pyrimidine bases linked by a phosphodiester backbone [59], [60]. It is remarkable that despite this simple chemistry, RNA can fold into complex three-dimensional structures that are capable of catalysis [61]–[63]. However, the simplicity of the RNA nucleotide “alphabet” is at the heart of the structural diversity of the suboptimal ensemble of structures [40], [43], [64]. Indeed, the limited base-pairing partners for any of the four bases makes it much more likely to find multiple complementary regions in a given RNA sequence [65].

To illustrate this concept, we roughly estimate that an RNA sequence longer than 314 nucleotides can adopt more conformations than there are atoms in the Universe (see methods). A consequence of this is the remarkable result observed when a program like Sfold (which performs Boltzmann sampling) is run twice in a row. The number of identical structures in the two suboptimal samplings of 1000 structures for the *same* RNA is usually between 2 to 3 for RNAs of moderate lengths [40] and can often be zero for long RNAs [43], [65]. The probability of the minimum free energy structure in the Boltzman ensemble is in fact negligible for most large RNAs [40], [66]. Our findings of high MI base-pairs inconsistent with a single secondary structure, but always found in the suboptimal ensemble, suggest that these alternative structures are tolerated by evolution. Given the difficulty of evolving an RNA to adopt a single conformation, it is likely that regulatory systems involving RNA have adapted to these alternative conformations and in some cases even selected for them. Adopting a single conformation is not necessarily a pre-requisite for biological function as long as a significant fraction of the RNAs do adopt the active conformation at any given time.

The functional role of alternative conformations in riboswitches is well established [9], [28]. The data we present in Figure 1 is consistent with at least two structures. Interestingly, Boltzmann sampling of the suboptimal ensemble of the purine riboswitch (Figure 2A) reveals three major conformations (blue, green and red). However, even this classification is somewhat of an oversimplification given that the first two principal components only capture a little more than a third of the structural complexity of the suboptimal ensemble. As such, evolving an RNA to adopt a single conformation represents a daunting task, even for an evolutionary process spanning billions of generations. Our data suggest that RNAs are evolved to adopt multiple conformations, even catalytic ribozymes.

The data presented in Figure 2C is particularly intriguing from an evolutionary perspective. Indeed, we find that highly related riboswitches seem to preserve ensemble partitioning. This is not *a priori* surprising, since ensemble partitioning is likely important to function in the cell. We and others have recently shown, however, that there are specific mutations in all RNAs that are highly disruptive to structure (in many cases these are disease-associated) and that these single point mutations affect ensemble partitioning [39], [67], [68]. The high degree of similarity in the different clades of riboswitches in terms of their ensemble partitioning (Figure 2C) suggests that evolution avoids these disruptive mutations. This is consistent with the importance of not only preserving the ability to adopt multiple conformations, but also avoiding deleterious mutations that disrupt it.

An important consideration in interpreting our data is the role of RNA co-transcription and kinetic traps in folding to an active conformation [69]. The binding of exogenous molecules (including RNA chaperones) can significantly impact folding outcome [7], [70]. Furthermore, post-transcriptional modifications of RNA will necessarily change the folding landscape [71], [72]. The sequence ultimately selected by the evolutionary process is therefore under many different forms of selective pressure. Our analysis suggests that alternative conformations are neither selected for or against, but these may just be a consequence of selecting for a sequence that has phenotypically advantageous co-transcriptional folding pathways. Our analysis is also based on a comparison of homologous sequences in a family of RNAs with the assumption that they all have similar functional roles. Some of the alternative conformations consistent with high-MI base-pairs may also be a result of conserved RNA scaffolds adopting alternative function.

The ability to adopt specific alternative conformations may confer significant evolutionary advantages to RNAs. Near isoenergetic conformations are ideal for ligand induced switching, since binding of a specific ligand can easily shift the ensemble partitioning. Catalytic ribozymes, on the other hand must adopt a single and specific conformation to carry out catalysis. However, a majority of ribozymes readily misfold and this suggests these molecules may also act as switches in the cell [6], [49], [73]. Indeed, RNA chaperones help resolve these misfolds in an ATP dependent manner, suggesting a possible bi-molecular regulatory switch [70]. The ability of RNA to adopt multiple alternative conformations may in fact confer a significant evolutionary advantage in terms of adaptability and ability to control regulatory networks. As such, it is not surprising to find RNA playing such a key role in the central dogma of biology.

## Methods

### Purine riboswitch analysis

The purine riboswitch alignment was obtained from the RFAM [34]–[37] database (http://rfam.sanger.ac.uk/family/RF00167). The alignment in only included nucleotide positions corresponding to the P1, P2 and P3 stems. Each sequence was therefore extended by 100 nucleotides in the 3′ direction in order to allow for the ability to search for terminator stems. The RNIE software package was used to scan each of the sequences for Rho-independent terminators [74]. Sequences without a predicted terminator were removed from the analysis. The 3′ alignment of the P1 stem was then folded with the 5′ region of the predicted terminators in RNAfold to search for potential anti-terminator pairs [58], [75]. Sequences without any predicted pairs were further removed from the analysis. This resulted in a final set of 246 sequences that were used for the co-variation analysis presented in Figure 1. All alignments used for the analysis in this paper are provided in the supplement in Stockholm file format [76], [77].

A simple sequence alignment strategy was not sufficient to correctly align the terminator stems in the purine riboswitch alignment. Alignments were thus adjusted to reflect the predicted terminator stems identified using by RNAfold minimum free energy predictions of each individual sequence [58]. There are 8 possible pairing positions in the P1 stem. The nucleotides within the 5′ predicted terminator were aligned according so as to correspond with the predicted base-pairs on the 3′ end of the P1 stem. The corresponding 3′ terminator nucleotides were then retrieved and aligned according to the original predicted terminator pairs. This results in the consensus terminator stem formed by 4 base-pairs shown in Figure 1.

### Additional alignments

Additional alignments used in this manuscript were retrieved either from the comparative RNA website hosted by the Gutell Lab at the University of Texas at Austin or the RFAM database [51]. The alignments were refined to the most mutationally diverse and gap limited sequences according to the reference sequence and structure. The final analysis included 1332 16S, 2204 23S, 246 purine riboswitch, 289 group I intron, 1642 Glycine (RF00504) and 601 GMP (RF01051) riboswitch sequences.

### Mutual information and consistency analysis

Mutual Information scores were calculated using the MIfold MATLAB package [18], [78]. The ‘M’ algorithm specified in the package was used to calculate the mutual information scores. This formula computes the score as the information content describing the degree to which the two positions in the alignment can or cannot form a base pair. Canonical and wobble base pairs are specified as the only pairs allowed in the algorithm. The inconsistency parameter specified is the percentage of non-allowable pairs for the indicated positions.

Respective ROC curves were generated by incrementally thresholding the mutual information scores for pairs in the accepted structure. True positives were established as base pairs in the accepted structure with an MI value above the threshold. False positives were base pairs not in the accepted structure that had an MI value above the threshold. False negatives were set as base pairs in the accepted structure with an MI value below the threshold. True negatives were base pairs not in the accepted structure that had an MI value below the threshold. The TPR, FPR and PPV were calculated as:
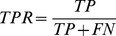
(1)

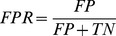
(2)

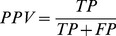
(3)


The RNAfold TPR and PPV values were generated by sampling pairs at varying MI cutoffs. All pairs with an MI value above the cutoff were sampled and a constraint file was created based on the pairs. The constraint file was used in a secondary structure prediction generated by RNAfold. For any one mutual information score, 100 constraints/predictions were made. The respective accepted structures were also used as constraints. Random pairs in the accepted structure were removed and the remaining pairs were used as a constraint for the RNAfold prediction. The predicted structures were then compared to the accepted structure.

### Figures and diagrams

Structure diagrams were made using both VARNA and the Circle Compare algorithm found in the RNAstructure software package [79]–[81]. Sequence identity displays are a subset of the full set of sequences used for the analysis and were made using Jalview. Principle component analysis of the suboptimal ensemble was carried out as previously described [39]. All calculations and graphs were done using R version 2.1.12 and Python 2.7.2.

### Size of the suboptimal ensemble estimation

We assumed that the number of possible RNA secondary structures can be estimated as increasing with 1.8^N^ (where N is the sequence length) and that there are approximately 10^80^ observable atoms in the universe [66], [82]. Thus, solving for N we estimate that an RNA molecule longer than 314 nucleotides (e.g. the *T. thermophila* group I intron) is able to adopt more conformations than there are atoms in the universe.

## Supporting Information

Dataset S1 Alignments in Stockholm (.sto) format for all RNAs analyzed in the manuscript compressed as a .zip archive.
(ZIP)

Figure S1 MI analysis of Watson-Crick base-pairing for the Purine Riboswitch alignment from RFAM, RF00167 (A) Circle diagram of the Adenine Riboswitch structure. The crystal structure is based on PDB ID 1Y26, base-pairs having MI support in the crystal structure above the specified threshold are colored red, while those in grey are below the threshold. Base-pairs for which there is MI support above the threshold but that are not in the accepted structure are shown in green. B) Sensitivity and PPV as a function of MI for the Purine Riboswitch alignment. In this case Sensitivity is computed as the number of “red” pairs divided by the total number in the accepted structure (gray pairs). PPV is computed as the number of True Positives (TP, in this case red pairs) divided by the sum of the TP and False Positives (FP, green pairs). C) Mean crystallographic distance in **Å** of non-accepted (green) base-pairs at the three MI thresholds. We see that at all three thresholds the means are above the mean of canonical base-pairs (shown as a red line at 13.6 **Å**), indicating that the co-variation is not likely the result of stabilizing an RNA tertiary contact.
(TIFF)

Figure S2 MI analysis of Watson-Crick base-pairing for the phenylalanine tRNA alignment from the Comparative RNA Website (A) Circle diagram of the phenylalanine tRNA structure. The crystal structure is based on PDB ID 1EHZ, base-pairs having MI support in the crystal structure above the specified threshold are colored red, while those in grey are below the threshold. Base-pairs for which there is MI support above the threshold but that are not in the accepted structure are shown in green. B) Sensitivity and PPV as a function of MI for the phenylalanine tRNA alignment. In this case Sensitivity is computed as the number of “red” pairs divided by the total number in the accepted structure (gray pairs). PPV is computed as the number of True Positives (TP, in this case red pairs) divided by the sum of the TP and False Positives (FP, green pairs). C) Mean crystallographic distance in **Å** of non-accepted (green) base-pairs at the three MI thresholds. We see that at all three thresholds the means are above the mean of canonical base-pairs (shown as a red line at 13.5 **Å**), indicating that the co-variation is not likely the result of stabilizing an RNA tertiary contact.
(TIFF)

Figure S3 MI analysis of Watson-Crick base-pairing for the 16s ribosomal alignment from the Comparative RNA Website (A) Circle diagram of the 16s ribosomal structure. The crystal structure is based on PDB ID 1FJG, base-pairs having MI support in the crystal structure above the specified threshold are colored red, while those in grey are below the threshold. Base-pairs for which there is MI support above the threshold but that are not in the accepted structure are shown in green. B) Sensitivity and PPV as a function of MI for the 16s ribosomal alignment. In this case Sensitivity is computed as the number of “red” pairs divided by the total number in the accepted structure (gray pairs). PPV is computed as the number of True Positives (TP, in this case red pairs) divided by the sum of the TP and False Positives (FP, green pairs). C) Mean crystallographic distance in **Å** of non-accepted (green) base-pairs at the three MI thresholds. We see that at all three thresholds the means are above the mean of canonical base-pairs (shown as a red line at 13.6 **Å**), indicating that the co-variation is not likely the result of stabilizing an RNA tertiary contact.
(TIFF)

Figure S4 MI analysis of Watson-Crick base-pairing for the Group II Intron alignment from RFAM, RF02001 (A) Circle diagram of the Group II Intron structure. The crystal structure is based on PDB ID 3G78, base-pairs having MI support in the crystal structure above the specified threshold are colored red, while those in grey are below the threshold. Base-pairs for which there is MI support above the threshold but that are not in the accepted structure are shown in green. B) Sensitivity and PPV as a function of MI for the group II intron alignment. In this case Sensitivity is computed as the number of “red” pairs divided by the total number in the accepted structure (gray pairs). PPV is computed as the number of True Positives (TP, in this case red pairs) divided by the sum of the TP and False Positives (FP, green pairs). C) Mean crystallographic distance in **Å** of non-accepted (green) base-pairs at the three MI thresholds. We see that at all three thresholds the means are above the mean of canonical base-pairs (shown as a red line at 13.5 **Å**), indicating that the co-variation is not likely the result of stabilizing an RNA tertiary contact.
(TIFF)

Figure S5 MI analysis of Watson-Crick base-pairing for the Glycine Riboswitch alignment from RFAM, RF00504 (A) Circle diagram of the Glycine Riboswitch structure. The crystal structure is based on PDB ID 3OX0, base-pairs having MI support in the crystal structure above the specified threshold are colored red, while those in grey are below the threshold. Base-pairs for which there is MI support above the threshold but that are not in the accepted structure are shown in green. B) Sensitivity and PPV as a function of MI for the glycine riboswitch alignment. In this case Sensitivity is computed as the number of “red” pairs divided by the total number in the accepted structure (gray pairs). PPV is computed as the number of True Positives (TP, in this case red pairs) divided by the sum of the TP and False Positives (FP, green pairs). C) Mean crystallographic distance in **Å** of non-accepted (green) base-pairs at the three MI thresholds. We see that at all three thresholds the means are above the mean of canonical base-pairs (shown as a red line at 13.6 **Å**), indicating that the co-variation is not likely the result of stabilizing an RNA tertiary contact.
(TIFF)

Figure S6 MI analysis of Watson-Crick base-pairing for the Cyclid-diGMP Riboswitch alignment from RFAM, RF01051 (A) Circle diagram of the Cyclid-diGMP Riboswitch structure. The crystal structure is based on PDB ID 3IRW, base-pairs having MI support in the crystal structure above the specified threshold are colored red, while those in grey are below the threshold. Base-pairs for which there is MI support above the threshold but that are not in the accepted structure are shown in green. B) Sensitivity and PPV as a function of MI for the Cyclid-diGMP Riboswitch alignment. In this case Sensitivity is computed as the number of “red” pairs divided by the total number in the accepted structure (gray pairs). PPV is computed as the number of True Positives (TP, in this case red pairs) divided by the sum of the TP and False Positives (FP, green pairs). C) Mean crystallographic distance in **Å** of non-accepted (green) base-pairs at the three MI thresholds. We see that at all three thresholds the means are above the mean of canonical base-pairs (shown as a red line at 13 **Å**), indicating that the co-variation is not likely the result of stabilizing an RNA tertiary contact.
(TIFF)

Figure S7 Scatter plot of MI and Inconsistency values for all pairs in the Group I intron alignment. We only considered pairs with MI values >0.05 for this analysis. We find that the MI and Inconsistency values are negatively correlated such that our analyses are generally co-variation metric agnostic. i.e. we still find multiple low inconsistency base-pairs that are not compatible with a single RNA structure.
(TIFF)

Table S1 MI and inconsistency scores for all pairs (in excel spreadsheet format) for Riboswitch illustrated in Figure 1.
(XLS)
