# Supplementary figures and images for: Evolutionary Evidence for Alternative Structure in RNA Sequence Co-variation
Source: PLoS Comput Biol. 2013 Jul 25;9(7):e1003152. doi: 10.1371/journal.pcbi.1003152 (PMC3723493; doi:10.1371/journal.pcbi.1003152)

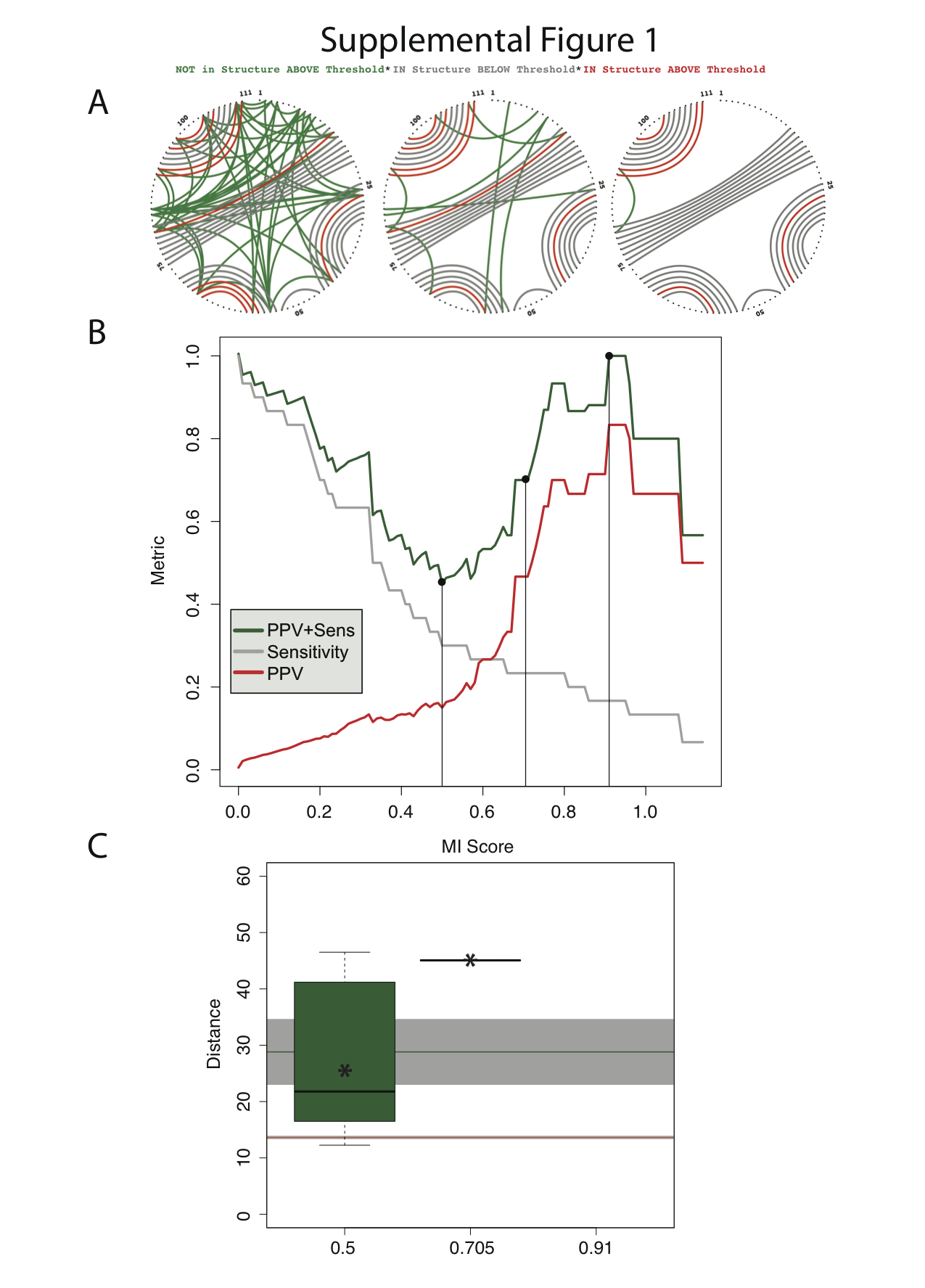

Supplement: Figure S1 — MI analysis of Watson-Crick base-pairing for the Purine Riboswitch alignment from RFAM, RF00167 (A) Circle diagram of the Adenine Riboswitch structure. The crystal structure is based on PDB ID 1Y26, base-pairs having MI support in the crystal structure above the specified threshold are colored red, while those in grey are below the threshold. Base-pairs for which there is MI support above the threshold but that are not in the accepted structure are shown in green. B) Sensitivity and PPV as a function of MI for the Purine Riboswitch alignment. In this case Sensitivity is computed as the number of “red” pairs divided by the total number in the accepted structure (gray pairs). PPV is computed as the number of True Positives (TP, in this case red pairs) divided by the sum of the TP and False Positives (FP, green pairs). C) Mean crystallographic distance in Å of non-accepted (green) base-pairs at the three MI thresholds. We see that at all three thresholds the means are above the mean of canonical base-pairs (shown as a red line at 13.6 Å), indicating that the co-variation is not likely the result of stabilizing an RNA tertiary contact. (TIFF) [file pcbi.1003152.s002.tiff]

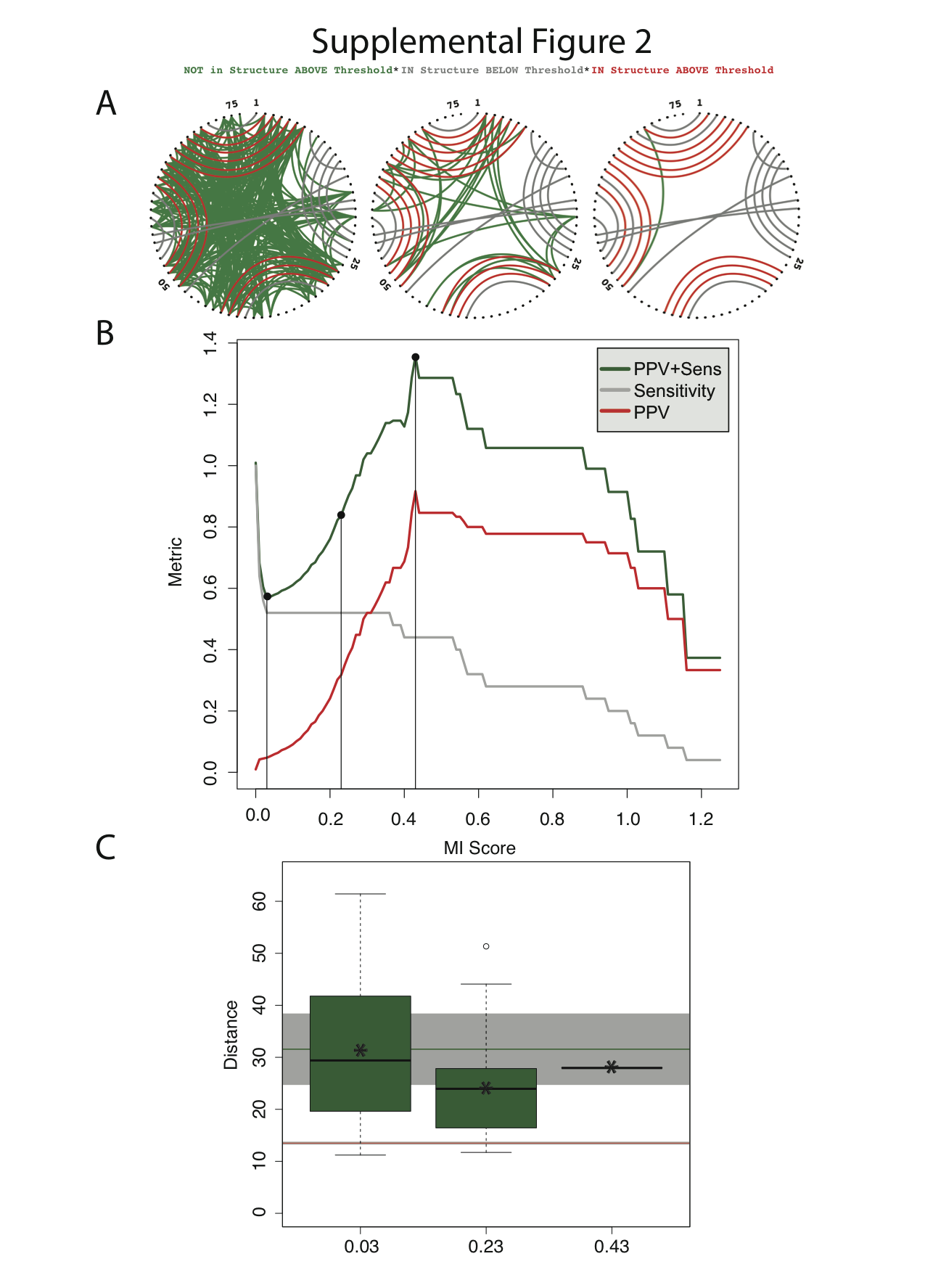

Supplement: Figure S2 — MI analysis of Watson-Crick base-pairing for the phenylalanine tRNA alignment from the Comparative RNA Website (A) Circle diagram of the phenylalanine tRNA structure. The crystal structure is based on PDB ID 1EHZ, base-pairs having MI support in the crystal structure above the specified threshold are colored red, while those in grey are below the threshold. Base-pairs for which there is MI support above the threshold but that are not in the accepted structure are shown in green. B) Sensitivity and PPV as a function of MI for the phenylalanine tRNA alignment. In this case Sensitivity is computed as the number of “red” pairs divided by the total number in the accepted structure (gray pairs). PPV is computed as the number of True Positives (TP, in this case red pairs) divided by the sum of the TP and False Positives (FP, green pairs). C) Mean crystallographic distance in Å of non-accepted (green) base-pairs at the three MI thresholds. We see that at all three thresholds the means are above the mean of canonical base-pairs (shown as a red line at 13.5 Å), indicating that the co-variation is not likely the result of stabilizing an RNA tertiary contact. (TIFF) [file pcbi.1003152.s003.tiff]

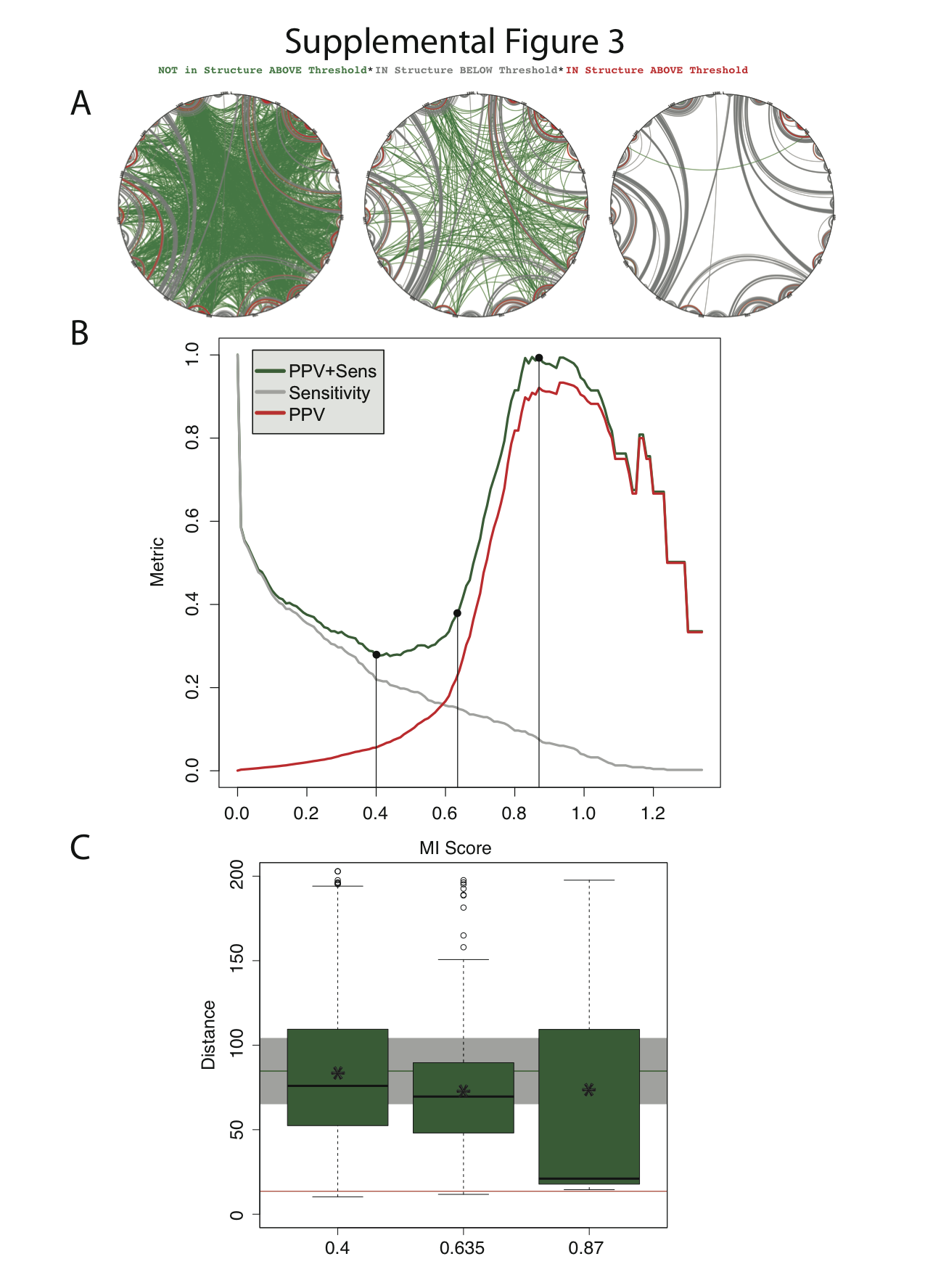

Supplement: Figure S3 — MI analysis of Watson-Crick base-pairing for the 16s ribosomal alignment from the Comparative RNA Website (A) Circle diagram of the 16s ribosomal structure. The crystal structure is based on PDB ID 1FJG, base-pairs having MI support in the crystal structure above the specified threshold are colored red, while those in grey are below the threshold. Base-pairs for which there is MI support above the threshold but that are not in the accepted structure are shown in green. B) Sensitivity and PPV as a function of MI for the 16s ribosomal alignment. In this case Sensitivity is computed as the number of “red” pairs divided by the total number in the accepted structure (gray pairs). PPV is computed as the number of True Positives (TP, in this case red pairs) divided by the sum of the TP and False Positives (FP, green pairs). C) Mean crystallographic distance in Å of non-accepted (green) base-pairs at the three MI thresholds. We see that at all three thresholds the means are above the mean of canonical base-pairs (shown as a red line at 13.6 Å), indicating that the co-variation is not likely the result of stabilizing an RNA tertiary contact. (TIFF) [file pcbi.1003152.s004.tiff]

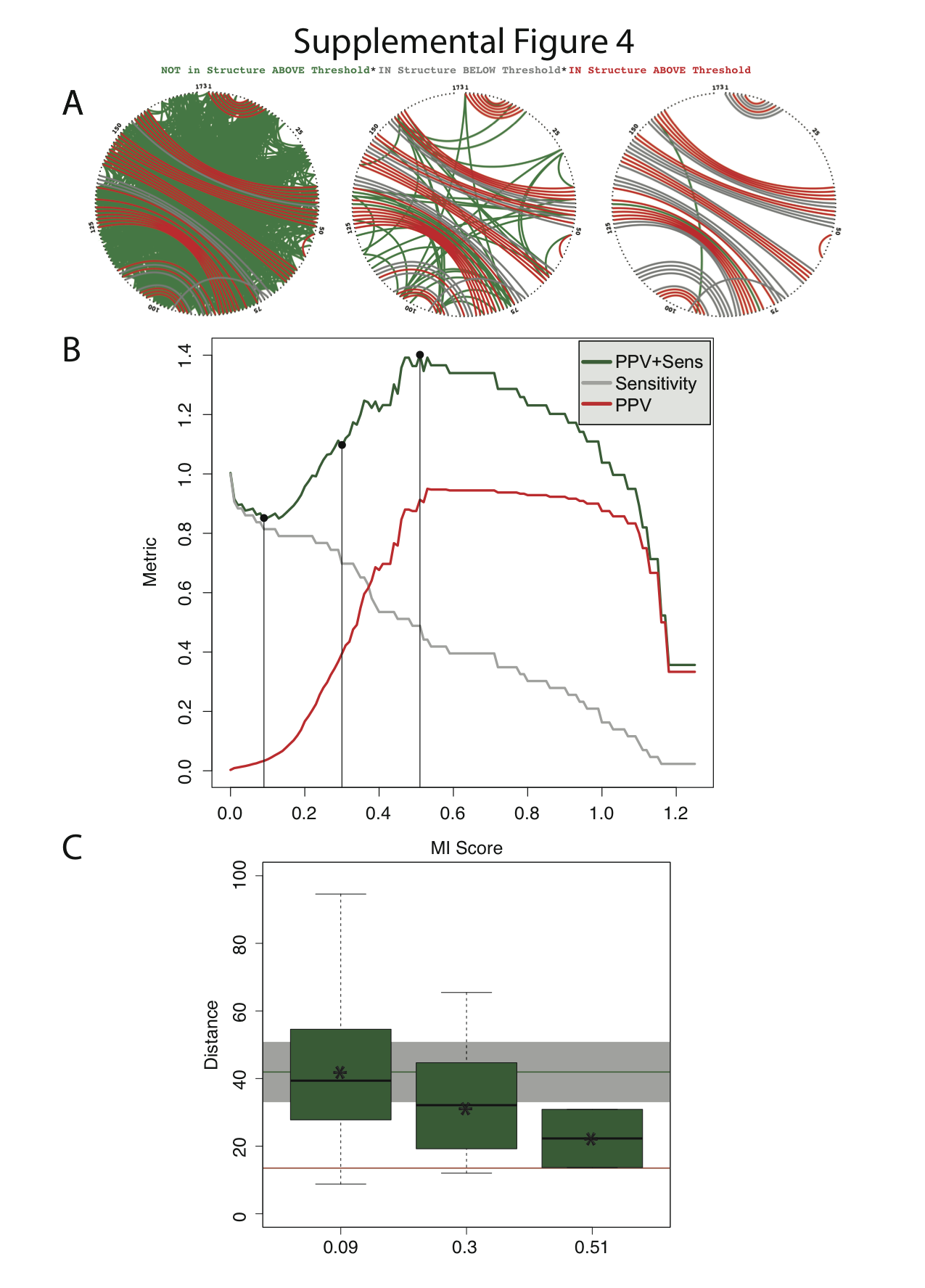

Supplement: Figure S4 — MI analysis of Watson-Crick base-pairing for the Group II Intron alignment from RFAM, RF02001 (A) Circle diagram of the Group II Intron structure. The crystal structure is based on PDB ID 3G78, base-pairs having MI support in the crystal structure above the specified threshold are colored red, while those in grey are below the threshold. Base-pairs for which there is MI support above the threshold but that are not in the accepted structure are shown in green. B) Sensitivity and PPV as a function of MI for the group II intron alignment. In this case Sensitivity is computed as the number of “red” pairs divided by the total number in the accepted structure (gray pairs). PPV is computed as the number of True Positives (TP, in this case red pairs) divided by the sum of the TP and False Positives (FP, green pairs). C) Mean crystallographic distance in Å of non-accepted (green) base-pairs at the three MI thresholds. We see that at all three thresholds the means are above the mean of canonical base-pairs (shown as a red line at 13.5 Å), indicating that the co-variation is not likely the result of stabilizing an RNA tertiary contact. (TIFF) [file pcbi.1003152.s005.tiff]

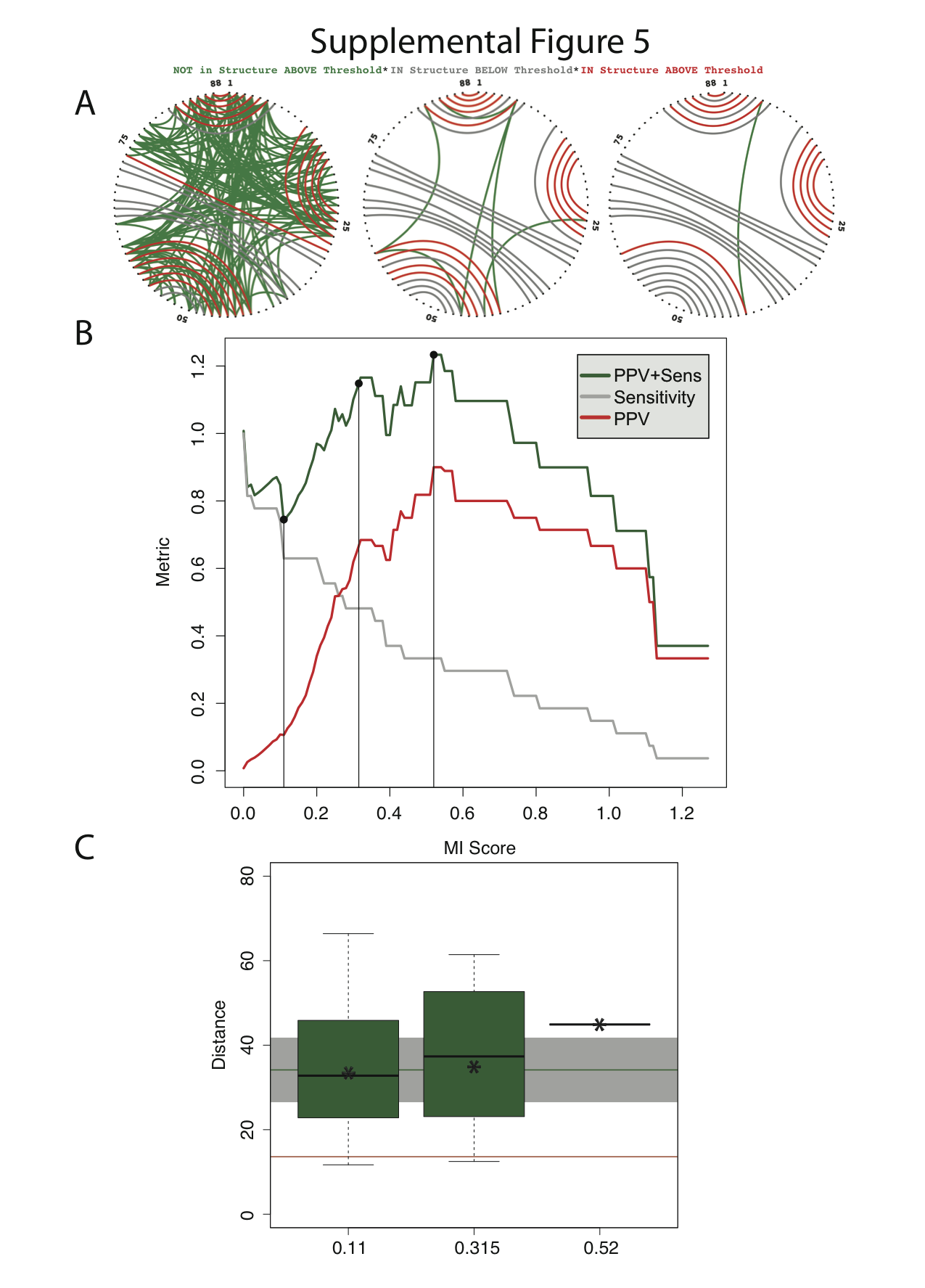

Supplement: Figure S5 — MI analysis of Watson-Crick base-pairing for the Glycine Riboswitch alignment from RFAM, RF00504 (A) Circle diagram of the Glycine Riboswitch structure. The crystal structure is based on PDB ID 3OX0, base-pairs having MI support in the crystal structure above the specified threshold are colored red, while those in grey are below the threshold. Base-pairs for which there is MI support above the threshold but that are not in the accepted structure are shown in green. B) Sensitivity and PPV as a function of MI for the glycine riboswitch alignment. In this case Sensitivity is computed as the number of “red” pairs divided by the total number in the accepted structure (gray pairs). PPV is computed as the number of True Positives (TP, in this case red pairs) divided by the sum of the TP and False Positives (FP, green pairs). C) Mean crystallographic distance in Å of non-accepted (green) base-pairs at the three MI thresholds. We see that at all three thresholds the means are above the mean of canonical base-pairs (shown as a red line at 13.6 Å), indicating that the co-variation is not likely the result of stabilizing an RNA tertiary contact. (TIFF) [file pcbi.1003152.s006.tiff]

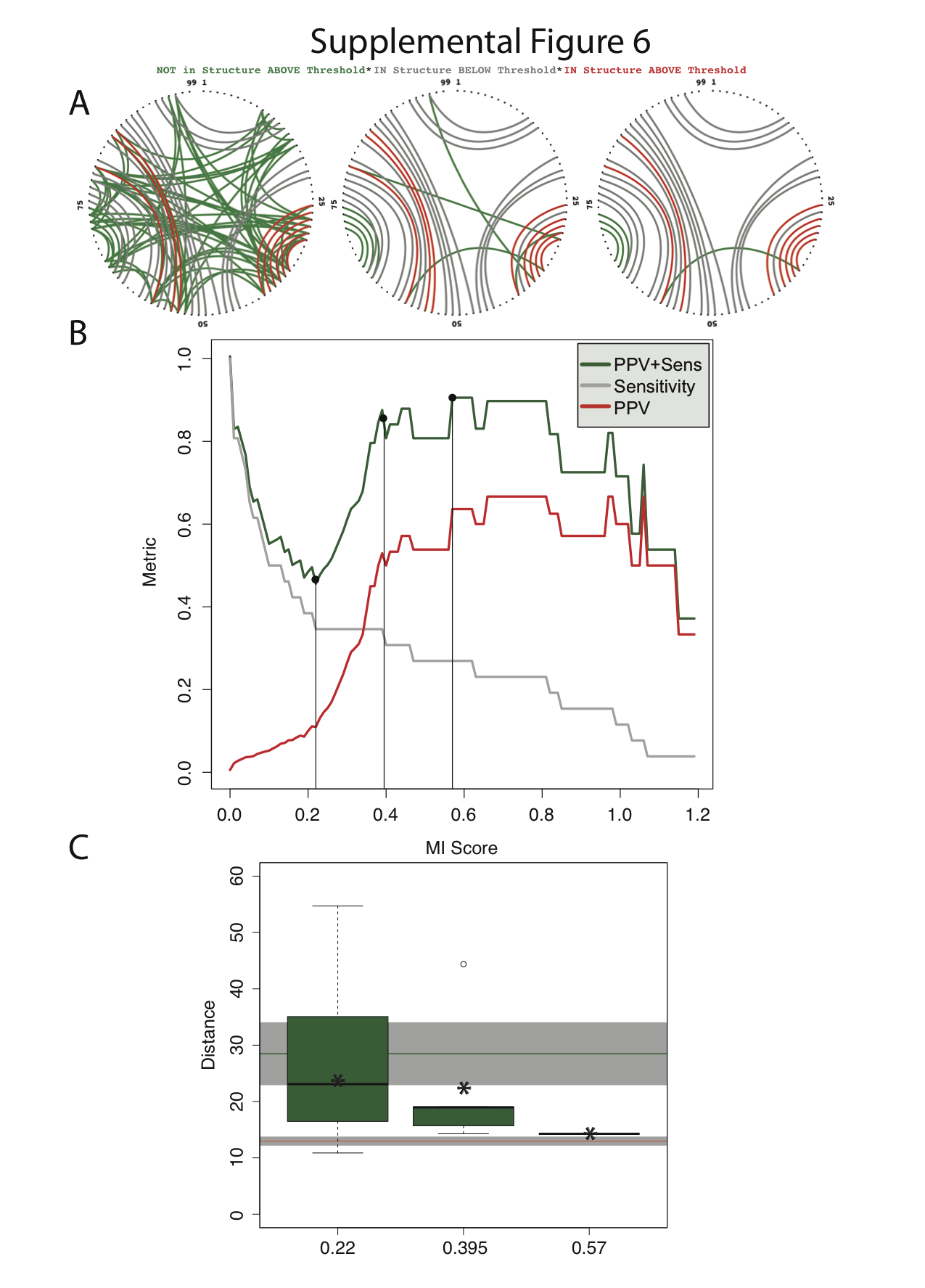

Supplement: Figure S6 — MI analysis of Watson-Crick base-pairing for the Cyclid-diGMP Riboswitch alignment from RFAM, RF01051 (A) Circle diagram of the Cyclid-diGMP Riboswitch structure. The crystal structure is based on PDB ID 3IRW, base-pairs having MI support in the crystal structure above the specified threshold are colored red, while those in grey are below the threshold. Base-pairs for which there is MI support above the threshold but that are not in the accepted structure are shown in green. B) Sensitivity and PPV as a function of MI for the Cyclid-diGMP Riboswitch alignment. In this case Sensitivity is computed as the number of “red” pairs divided by the total number in the accepted structure (gray pairs). PPV is computed as the number of True Positives (TP, in this case red pairs) divided by the sum of the TP and False Positives (FP, green pairs). C) Mean crystallographic distance in Å of non-accepted (green) base-pairs at the three MI thresholds. We see that at all three thresholds the means are above the mean of canonical base-pairs (shown as a red line at 13 Å), indicating that the co-variation is not likely the result of stabilizing an RNA tertiary contact. (TIFF) [file pcbi.1003152.s007.tiff]

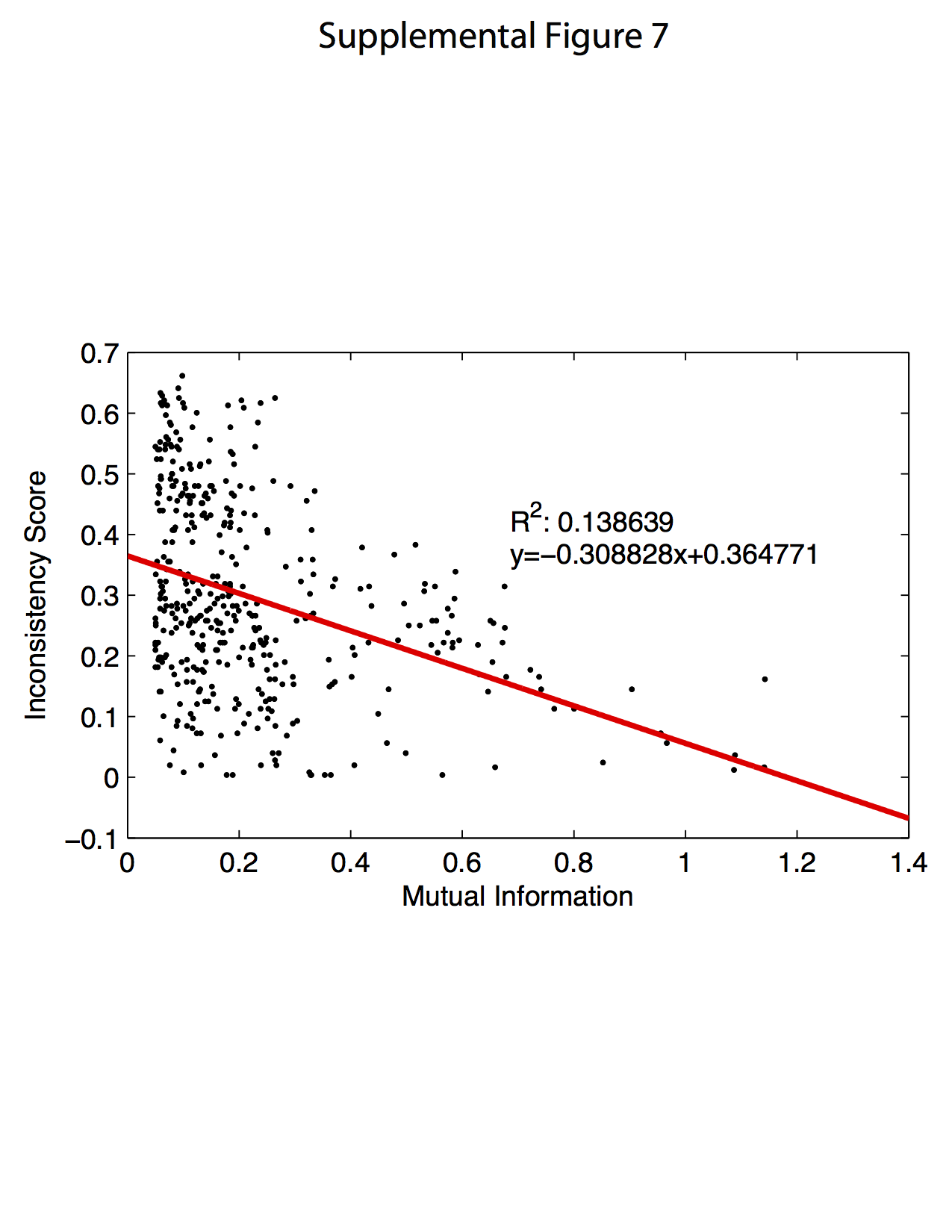

Supplement: Figure S7 — Scatter plot of MI and Inconsistency values for all pairs in the Group I intron alignment. We only considered pairs with MI values >0.05 for this analysis. We find that the MI and Inconsistency values are negatively correlated such that our analyses are generally co-variation metric agnostic. i.e. we still find multiple low inconsistency base-pairs that are not compatible with a single RNA structure. (TIFF) [file pcbi.1003152.s008.tiff]
